# Polysaccharide Hydrogels with Waste Wool Fibre as Matrix for Potential Use as CRF Fertiliser

**DOI:** 10.3390/molecules30132885

**Published:** 2025-07-07

**Authors:** Ewa Szczepanik, Edyta Molik, Kinga Pielichowska

**Affiliations:** 1 Department of Glass Technology and Amorphous Coatings, Faculty of Materials Science and Ceramics, AGH University of Krakow, Al. Mickiewicza 30, 30-059 Krakow, Poland; eszczepanik@agh.edu.pl; 2 Department of Animal Biotechnology, Faculty of Animal Science, University of Agriculture in Krakow, Al. Mickiewicza 24/28, 31-059 Krakow, Poland; rzmolik@cyf-kr.edu.pl

**Keywords:** wool, natural fibres, hydrogels, controlled-release fertilisers (CRFs)

## Abstract

At a time of climate change, farmers face difficulties in providing food for a growing population. This results in the overuse of water and fertilisers. The aim of the research was to test the possibility of introducing waste sheep wool fibres into a hydrogel to obtain a stable material that could improve water retention and could serve as a fertiliser material matrix. Wool fibres and hydrogel were chosen because of their ability to store water and their degradability. An evaluation of the swelling degree of different alginate-based hydrogel matrices was performed to select the matrix. The stability and water bonding of hydrogels with different wool fibre content were analysed and evaluated by thermogravimetric analysis (TGA) and differential scanning calorimetry (DSC). The microstructure and the effect of fibres on the uniformity of the hydrogel were assessed using SEM and optical microscopy. The degree of water retention in the soil was also evaluated. The results showed that it is possible to incorporate wool fibres into the hydrogel matrix and the wool fibres make the composite porous, which allows water penetration into the material much more easily. This research has shown the possibility of using waste wool fibres as an active ingredient in sustainable fertiliser materials.

## 1. Introduction

Recent years have been marked by the fight against climate change and the problems it brings. One of the areas heavily affected by the natural environment is agriculture. The ever-growing number of people increases the demand for food, leading to the use of large amounts of fertilisers and water. By 2050, population growth is estimated to result in the need to increase crop production as much as 100% compared to the early 2000s [1]. Difficult weather conditions, such as droughts, require proper management of water resources and maximum use of various solutions to avoid additional irrigation in fields with decreasing annual rainfall [2]. This involves an interdisciplinary approach to agriculture, including fertiliser production and operation [3]. Fertilisers are a group of key materials for agriculture, with a market value of almost USD 200 billion in 2022 [4]. Their production technologies and composition need to be continually improved to reduce their environmental impact [5]. With all the problems mentioned, it is crucial for modern engineering of fertiliser materials to use environmentally neutral resources and to be able to introduce a controlled release of nutrients and improved water retention in the soil.

In the search for solutions to improve water retention, superabsorbent materials such as hydrogels stand out. They can absorb water more than 300 times their own weight [6]. It is this characteristic that ensures that, after rain or irrigation, the water-holding capacity of the soil with hydrogels will increase. Hydrogels are a group of polymers that are joined by a 3D network, making them capable of repeatedly swelling and releasing water [7]. This feature of hydrogels is crucial in their use as fertiliser material in agriculture, because in addition to reducing the need for soil irrigation with the release of stored water in them, the release of nutrients into the environment may be possible [8]. Due to ecological and environmental limitations, biodegradable natural-derived polymers are much more popular now, because they limit soil pollution during degradation. It is possible to use only biodegradable hydrogels, as well as their combinations with synthetic polymers, which allows us to precisely determine the working range of the material and adjust fertiliser additives [9,10]. In addition to biodegradability, natural hydrogels have many advantages, such as low price and high availability [11]. Many natural polymers are being studied for agricultural applications—chitosan [9,10], gellan gum [7,12], starch [7], sodium alginate [8,11,13]. Sodium alginate (SA) belongs to the group of polysaccharides soluble in water, but forms a stable insoluble structure after cross-linking [12]. SA extracted from brown algae was initially used as an additive in food, and now due to its biocompatibility and ability to encapsulate substances (nutrients, drugs), it has found a very wide field of applications in medicine, industry, and agriculture [13].

An interesting aspect in the design of fertiliser materials is the use of various types of bio-waste, because they often contain plant nutrients, and in addition, the use of waste can reduce the cost of fertiliser production [14,15]. Research has focused on combinations of biodegradable hydrogels with residues such as cellulose, keratin, and vegetable waste, which fits in with sustainability goals of responsible consumption and production [16,17,18]. Keratin waste, including sheep wool, is being studied due to its nitrogen content, which is crucial for plant growth and may provide an additional natural source of this ingredient [18,19]. Sheep wool outside the textile industry is difficult to manage and disposal problems are raised from both an economic and livestock point of view [5,20]. Low-quality wool and short fibres are considered waste, yet they are rich in keratin. The fibres contain up to 20% nitrogen and 3–4% sulphur, which are essential nutrients for crops [21]. An additional advantage of cleaned wool is its ability to absorb moisture up to 33% by weight [5]. All these characteristics mean that degrading wool fibre can improve soil water retention and gradually, slowly release nitrogen, making it an interesting component of slow nutrient release fertilisers. In the European Union alone, annual wool production is more than 200,000 tonnes and this is mainly poor-quality coarse wool, which needs to be properly managed to avoid costly disposal [22].

The use of wool in agriculture has already been considered in various studies. Wool was considered a substrate in the hydroponic cultivation of cucumbers [23], where it was found to reduce water consumption by 13% per biomass yield, and a reduction in the greenhouse effect was determined. In a study by Broda et al. [24,25], raw wool was cut and placed in the soil for two seasons. Nitrogen compounds were found to be gradually released into the soil and this was correlated with the degree of fibre degradation. Nitrogen released improved the colour and height of the wheat and, consequently, improved the yield. Haque and Naebe [25] focused on comparing the way wool is applied to the soil by powder or pellet. The study showed that powder is comparable to traditional fertilisers, but the amount of wool should be adjusted to the appropriate soil moisture to balance retention ability. Sheep wool was also tested as an additive in spinach and tomato farming [26], where the wool pellet showed very similar properties to traditional fertiliser and was able to replace it. Abdallah et al. [27] analysed wool waste before and after carbonisation. The mixture of wool waste with soil proved to be beneficial for plant growth and biomass production. The addition of wool did not require additional mineral nitrogen fertilisation and improved soil moisture.

The focus of the present study was to investigate the possibility of combining hydrogels made of sodium alginate as widely studied materials for specialised fertilisers with waste wool fibre, which has interesting properties for plants because of its composition and water absorption capacity. The aim of the research was to test the possibility of producing a composite that could improve water retention, consisting of a hydrogel matrix and a wool additive capable of storing water.

## 2. Results and Discussion

### 2.1. Analysis of the Swelling Degree

**Figure 1:**
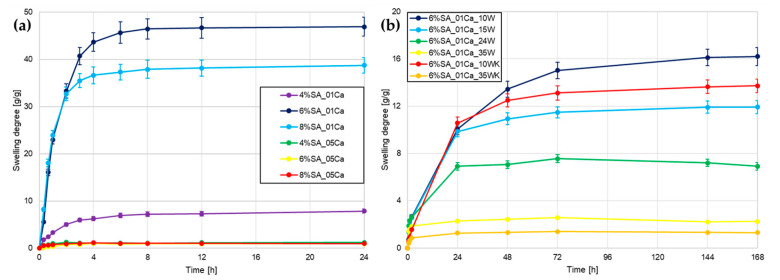
Swelling degree curves for different hydrogels: (**a**) hydrogel matrices; (**b**) SA hydrogel/wool composites.

The first test performed to assess water absorption capacity was to measure the degree of swelling. The study examined three different concentrations of SA (4, 6, and 8%) and two concentrations of cross-linker—0.1 and 0.5 M. From Figure 1a, it can be seen that it was immediately noticeable that hydrogels cross-linked with 0.5 CaCl_2_ did not swell back after drying, which ruled them out as a material for agricultural use. In all cases, the combination of SA with 0.5 M CaCl_2_ forms a very dense network, which is not optimal for hydrogels as they are unable to absorb large amounts of water after drying. For the 0.1 M cross-linker solution, the hydrogel with a low polymer concentration (4%) was also strongly cross-linked despite a 5-times lower CaCl_2_ concentration, as it significantly stands apart from hydrogels with 6 and 8% concentrations.

Based on the analysis performed, a 6% SA concentration with 0.1 M cross-linker was chosen for the addition of wool fibres, so that a comparison could be made as to whether the addition of absorbent fibres would improve an already very good working and water-absorbing material. Wool was added to SA at different concentrations (10, 15, 24, and 35%) so as to maintain a 6% concentration of the whole hydrogel. Two types of wool milling were tested and, in the case of cryogenic milling, the two extreme concentrations of fibre addition were chosen. During a longer swelling test shown in Figure 1b, hydrogels with a lot of wool slowly swelled in an aqueous environment. The more fibre, the less the hydrogel swelled. The fibres themselves absorbed water, but the expansion of the polymer network was greatly impaired by fibre incorporation. Short fibres milled in liquid nitrogen slightly worsened the swelling ability, but further tests are needed to confirm any differences.

Alginate-based hydrogels swell significantly in the first few hours and remain stable in weight after 24–48 h [11]. Hydrogel swelling is inherently linked to their porosity, as confirmed by Niu et al. [6]. Appropriate selection of the cross-linker and the concentration of the hydrogel itself made it possible to achieve water absorption several hundred times the weight of the dry hydrogel. This indicates that it is possible that using an even more dilute solution of CaCl_2_ would improve the properties of the hydrogel matrix itself. In the case of the study by Bora et al. [15], similarly to here, the large addition of cellulosic waste caused a decrease in the swelling capacity of the hydrogel, which resulted from the disruption of the network structure and additional cross-linking. A study by Merino et al. [16] also found that the addition of agricultural waste reduces swelling in water due to the effect of the additive on the formation of the hydrogel structure.

### 2.2. Microscopic and SEM Observations

**Figure 2:**
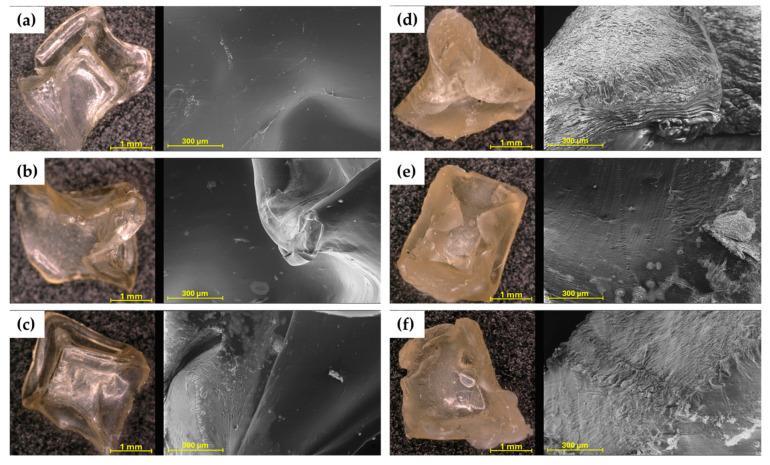
Optical microscope and SEM pictures for: (**a**) 4%SA_01Ca; (**b**) 6%SA_01Ca; (**c**) 8%SA_01Ca; (**d**) 4%SA_05Ca; (**e**) 6%SA_05Ca; (**f**) 8%SA_05Ca.

The microscopic images (Figure 2) immediately show the difference in colour and shape between hydrogels cross-linked with 0.1 and 0.5 M CaCl_2_ solution. The hydrogels 4%SA_05Ca, 6%SA_05Ca, and 8%SA_05Ca are less transparent and their shape was strongly deformed during drying. SEM observation confirmed all previous findings of poor swelling in these hydrogels. Figure 2d–f show a collapsed structure and compacted layers of the hydrogel network. Such a compressed structure could have made it difficult for water to enter during swelling. Compared to them, hydrogels cross-linked with 0.1 M CaCl_2_ in the images come out smooth with an even structure.

Analysis of the structure of hydrogels intended for agriculture using SEM allows for linking their effectiveness to the manufacturing process. In the study by Tiamwong et al. [11], SEM observation of SA hydrogel granules has shown that it is possible to control the smoothness of the surface through the method of manufacture and that phosphorus compounds can be incorporated into the network, which is beneficial for agricultural applications. Tests by Das et al. [17] also showed that hydrogels with low cross-linker concentration present a more uniform structure and, with increasing cross-linker concentration, the pore size, crucial in the swelling process, decreased.

**Figure 3:**
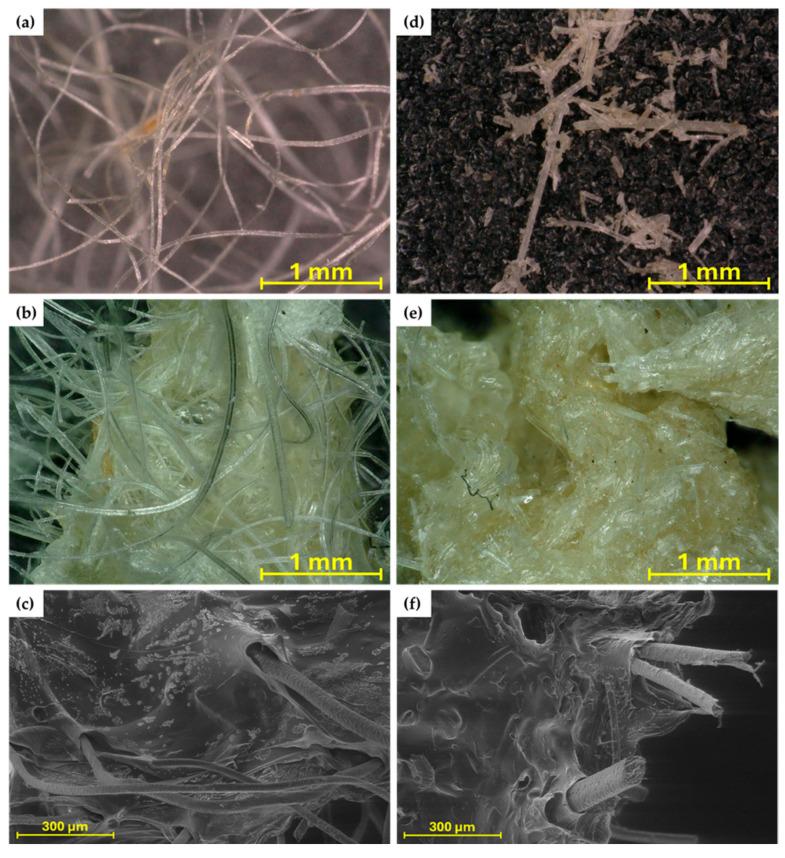
Microscopic images of traditionally milled wool (**a**), SA hydrogel/traditionally milled wool composite (6%SA_01Ca_35W) (**b**), SEM image of 6%SA_01Ca_35W (**c**), cryogenically milled wool (**d**), SA hydrogel/cryogenically milled wool composite (6%SA_01Ca_35WK) (**e**), and SEM image of 6%SA_01Ca_35W (**f**).

Sheep wool used in studies can be seen in Figure 3a,d. Figure 3b,e show hydrogels made with two types of wool. The hydrogel with the addition of cryogenically milled wool (Figure 3e) has a more compact texture due to the size of the micrometric fibres and the individual fibres do not extend beyond the hydrogel matrix.

SEM imaging clearly identified the effect of the addition of keratin fibres to the hydrogel (Figure 3c,f). These fibres have a characteristic shape and significantly disrupt the smooth microstructure of the hydrogel, causing a large number of open spaces and cracks to form, which can make it easier for water to penetrate the material, and, on the other hand, the hydrogel network responsible for its multiple swelling is disrupted.

The characteristic structure of the fibre and its composition have been demonstrated in many studies. SEM imaging has been used to confirm both the composition and degradation of wool fibre when placed in soil [28]. Fibres degrade unevenly, and studies indicate that they release elements such as nitrogen and phosphorus that are crucial for plant growth. The presence of such large and voluminous fibres in the polymer matrix results in the appearance of many empty spaces and cracks, which can lead to accelerated degradation, if only by hydrolysis [20].

### 2.3. Thermogravimetric Analysis

**Figure 4:**
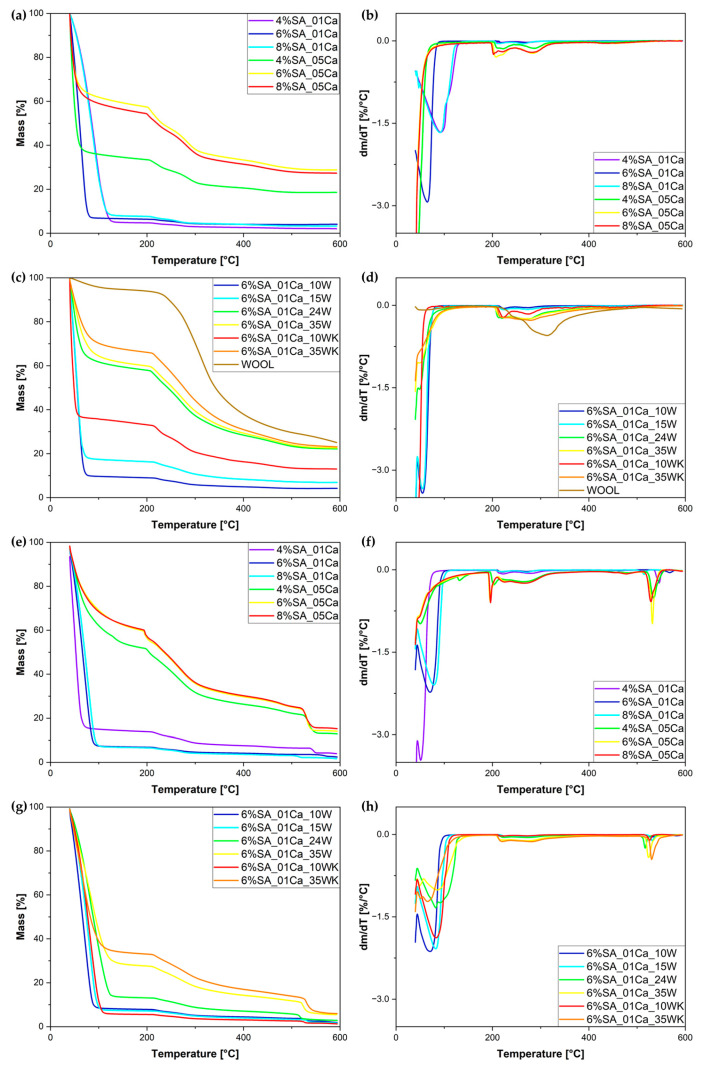
TG (**a**,**c**,**e**,**g**) and DTG (**b**,**d**,**f**,**h**) curves for obtained hydrogel matrix under nitrogen atmosphere (**a**,**b**), SA hydrogel/wool composites under nitrogen atmosphere (**c**,**d**), hydrogel matrix under air atmosphere (**e**,**f**), and SA hydrogel/wool composites under air atmosphere (**g**,**h**).

The same hydrogels, even before the drying process, were tested by thermogravimetric analysis (TG). The TG and DTG curves are shown in Figure 4. It can be seen, as in the SEM analysis, that cross-linking with a 0.5 M solution makes the structure very dense; little water is absorbed. This can be shown by the significantly lower amount of evaporated water for hydrogels cross-linked with a 0.5 M solution and the significantly higher residue at 600 °C (around 20%), whereas for hydrogels cross-linked with a 0.1 M solution most of the mass loss is attributed to water evaporation and only up to 4% of the mass is left at 600 °C. The results of the swelling test coincide with the curves obtained during the TG analysis (Figure 4c,d). The more wool, the greater the residual char amount. The DTG curve (Figure 4d) shows that the weight loss of swollen hydrogels occurs in two stages—first up to approximately 100 °C and a second stage beginning at approximately 200 °C.

Table 1 summarises the dynamics of evaporation of water from the hydrogel matrix. The study distinguishes between less and more cross-linked hydrogels, which are associated with a significant difference in the concentrations of the cross-linking solution. Hydrogels cross-linked with a 0.1 M solution are less cross-linked, while those cross-linked with a 0.5 M solution formed significantly more bonds, which caused the structure to become tightly packed during drying. Water escapes much more easily from less cross-linked hydrogels, as can be seen from the lower temperatures of initial mass loss (1 and 3%). This is due to the uncompressed and not overly cross-linked structure. For both concentrations of the cross-linker, it was the 4% polymer solution that left the least residual char, due to the low concentration of SA. In hydrogels, the first degradation process is water evaporation, which starts below 100 °C and is associated with the loss of water [29]. TG studies can serve as an element in the assessment of hydrogel water absorption, taking into account that evaporation of the water, due to its binding in the hydrogel, occurs in two stages and lasts up to approximately 150 °C [30,31]. The second mass loss at ca. 200 °C and higher is connected to the thermal degradation of the SA matrix [16,32].

In the same manner, Table 2 shows the stages of material mass loss for SA hydrogel/wool composites. With the addition of wool, evaporation occurs at slightly higher temperatures, most likely as a result of disturbed evaporation from within the structure destroyed by the addition of wool. Hydrogels with a large addition of wool (24 and 35%) absorbed little water during swelling—hence the 50% weight loss occurs at much higher temperatures [16,32]. Despite drying out, the wool still absorbed a small amount of moisture from the air, hence the initial drop in the TG curve. The most intensive degradation of the fibre occurs slightly later than that of the hydrogel matrix. Thermogravimetric analysis in an air atmosphere was performed for both matrices and hydrogel composites. For all samples, a second stage of degradation was observed at temperatures around 550 °C. In the case of 0.5 M cross-linked matrices, a lower proportion of water evaporation was again observed, and the residue, due to the higher amount of calcium in the structure, is significantly higher. In the case of hydrogel composites, it can be seen that the residue is significantly lower than in an inert atmosphere and in most cases is below 2%, but for a large addition of traditionally milled fibres, the residue is almost three times higher, which may be due to the characteristics of long fibres.

Thermal analysis studies on hydrogels with cellulose waste additives confirmed the high stability of the materials and the effect of the additive on the structure of the pure hydrogel [15]. In studies by Merino et al. [16], degradation up to 300 °C is first attributed to evaporation and then to polysaccharide degradation; hence, there is increasing residue due to the addition of wool fibres in the samples presented.

### 2.4. Differential Scanning Calorimetry Analysis

**Figure 5:**
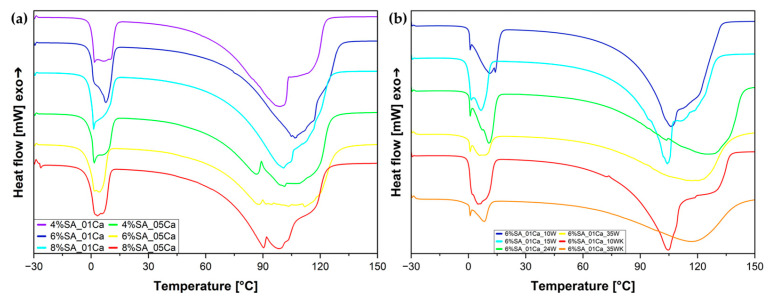
DSC curves for SA hydrogel matrix (**a**) and SA hydrogel/wool composites (**b**).

A differential scanning calorimetry (DSC) analysis was used to assess the states in which water binds with the hydrogel structure. The results for the hydrogels after fabrication and before the drying process are shown in Figure 5a. During the melting of the frozen water near 0 °C, a characteristic curve can be observed, consisting of two overlapping peaks corresponding to freezing bound water and free water. There is also the third state, which is non-freezing-bound water [7]. Furthermore, it can be seen from the DSC curves that evaporation proceeds in a disturbed manner because of the escape of water from within the highly porous structures and, in the case of hydrogels cross-linked with concentrated solution, also from the compacted structure. The DSC test (Figure 5b) for the wool hydrogels was carried out after the swelling process, to clearly relate the water states to the water absorbed during swelling and to simulate the performance of the dry material after placement in the soil and subsequent irrigation. Based on the peak resulting from the melting of water at around 0 °C, it can be seen that melting occurs in stages and the total heat of fusion is the result of the combined effect of two states of water—weakly bound water and free water. Interestingly, for hydrogels with a large amount of wool (24 and 35%), the evaporation process is delayed and reaches its maximum at higher temperatures—it is possible that the large amount of fibres blocks the hydrogel network, and the water has to evaporate through the fibres.

Table 3 shows more precise results from which the water states in the produced hydrogel matrices can be assessed. From the swelling test and the DSC curve analysis, the amount of water in the hydrogel and the freezing and non-freezing water can be calculated. For hydrogels with 0.5 M cross-linker, no further calculations were performed because of their inability to swell again. The hydrogel with 6% SA concentration has the most freezing water in its structure, which should allow it to absorb and release water easily (possibility of multiple cycles in the soil). This hydrogel also showed the highest absorption during swelling.

In the study, Sabadini et al. [7] investigated the effect of cross-linking on water status. Less cross-linked hydrogels are characterised, as in the studies presented above, by a higher amount of freezing water. Guan et al. [33] showed that water first binds to the structure and only from a certain degree of swelling begins to be visible in the DSC curves, which, in the case of the developed hydrogels, may indicate saturation of their structure with bound water.

An analysis of the water states in SA hydrogel/wool composites based on the melting curve is shown in Table 4. For a large fibre addition (35%), the total amount of water is less than 80%, but there is much more strongly bound non-freezing water in the structure, which can be related to the water deep in the structure absorbed by the fibres, which are reluctant to give it up or do so much more slowly than the hydrogel itself. Only when 15% of traditionally milled fibre is added does the amount of bound water increase strongly in relation to pure hydrogel; in the case of finer milled wool, this effect is noticeable for as little as 10%. Most likely, this is due to the greater surface area of the fibre available to absorb water.

In studies of hydrogel fibres with cotton [34], a shift in the peak of water evaporation was also observed when the fibres were added to the hydrogel, which may be due to hydrogen bonds between the fibre and the hydrogel matrix. Studies also mark increased structural stability after the introduction of fibre additives [15,17,35]. The shift in water evaporation and thus a more compact structure were also shown here by the hydrogels tested with a large addition of wool.

**Figure 6:**
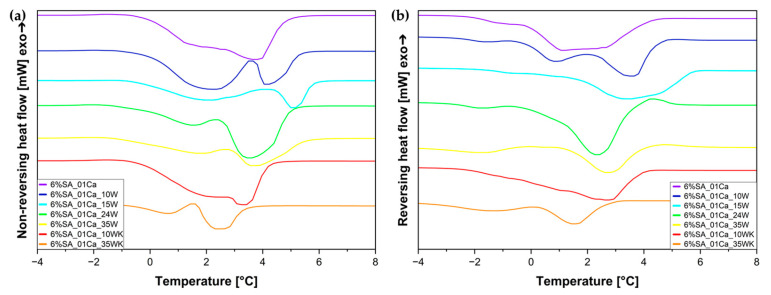
Temperature-modulated DSC (TOPEM DSC) curves for SA hydrogels/wool composite: (**a**) non-reversing heat flow; (**b**) reversing heat flow.

To confirm the specific nature of water in hydrogel composites, a temperature-modulated DSC analysis was also performed (Figure 6). For both reversing and non-reversing heat flow, two peaks each can be seen. Non-reversing heat flow can be matched to irreversible evaporation of water from the hydrogel (mainly weakly and unbound water), and the small peaks in the reversing heat flow diagram (Figure 6b) can come from the remodelling of the structure of strongly bound water in the hydrogel.

### 2.5. Water Retention Study

**Figure 7:**
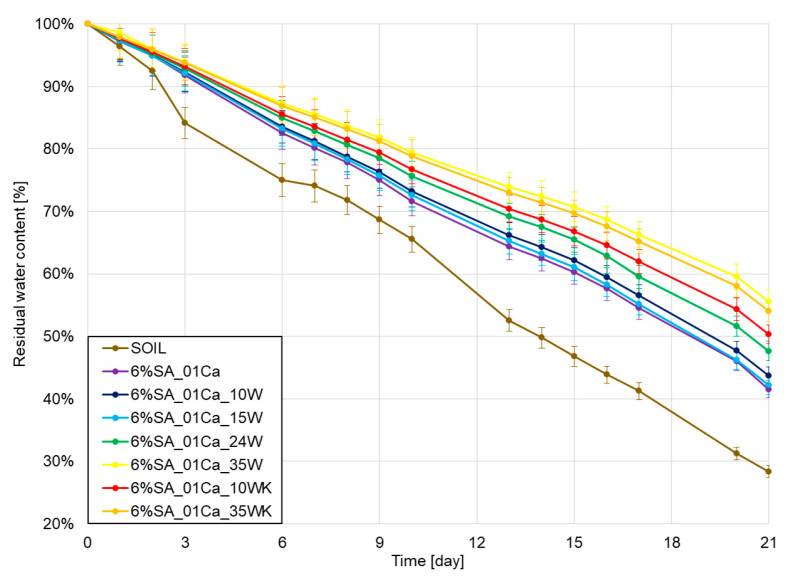
Water retention test results for SA hydrogels/wool composite.

In the very end, water retention was checked under laboratory conditions in the soil over a period of three weeks, and the results of water loss are shown in Figure 7. As expected, all hydrogels improved retention in relation to the soil. With a small addition of wool, no differences were observed with respect to the hydrogel alone, but it should be remembered that in this case the addition of wool may still be beneficial due to the incorporation of the waste and the addition of a natural nitrogen source. A large amount of wool further improves water retention relative to that of the hydrogel alone, despite a weaker degree of swelling in aqueous environments. This may be due to the observed more difficult evaporation from the deformed structure of the hydrogel as well as the fibre itself.

Hydrogel studies using keratin waste have shown the good properties of these compounds in improving water retention [18]. The addition of 1–5% hydrogel to the soil improved the soil’s water release rate several times and differences were noticeable even after 120 days. In studies, most hydrogels improve water retention capacity by several percent [36], even despite swelling at only 10–15 times the weight of the hydrogel. The large addition of wool introduced in this study increased this value even further by a few percent and showed that the combination of fibre and hydrogel is beneficial for soil moisture retention.

Studies have shown that the addition of sheep’s wool does not adversely affect phytotoxicity and, in addition, improves plant growth [27]. Hydrogels are a widely studied group of materials for agriculture that have a positive impact on crop yields and heights [37]. The combination of these two types of material can have both a positive effect on water retention through the use of hydrogel, an extended release time through the complex fibre structure, and additional beneficial nitrogen release. Furthermore, the introduction of waste fibres will have a positive impact on their environmental footprint [19] and the value of the material in large-scale applications.

## 3. Materials and Methods

The materials used in this work are hydrogels based on sodium alginate (viscosity = 1000 to 1500 mPa’s in 1% solution, ThermoFisher Scientific, Waltham, MA, USA) with the addition of wool cross-linked with a solution of CaCl_2_ (anhydrous, p.a., Chempur, Piekary Śląskie, Poland). Mixed sheep wool was supplied from the Experimental Station in Bielany by the University of Agriculture in Krakow.

In the first part of the study, hydrogels of different concentrations were made and cross-linked with 0.1 M or 0.5 M CaCl_2_ solutions. A summary of the materials made is summarised in Table 5. The process for each was as follows: an appropriate amount of sodium alginate (SA) powder was mixed with water to produce 30 g of SA solution. Then, the material was immersed in 30 mL of CaCl_2_ solution and left to cross-link for 48 h in wide flat-bottomed plastic tubes. The material was then cut into 5 mm cubes and dried in a laboratory oven at 50 °C.

The second part of the study was conducted on hydrogels containing wool fibre. The raw fibre was washed with water and hard soap and then dried for 48 h in a laboratory dryer at 50 °C. The pure fibres were shredded in a cutting mill (Fritsch Pulverisette 15, Fritsch, Idar-Oberstein, Germany). Some of the fibres were further processed using cryogenic milling in liquid nitrogen (Cole-Parmer SamplePrep CG-400 Freezer/Mill, Cole-Parmer, Vernon Hills, IL, USA). The fibres were put into a dedicated tube immersed in liquid nitrogen and subjected to a triple cycle of 2 min freezing/3 min milling.

The SA hydrogel/wool composites were prepared in the same way as described above, except that the sodium alginate solution was also mixed with an appropriate amount of wool fibre to obtain the chosen gel concentration of gel and then immersed in the CaCl_2_ solution. The other stages proceeded in the same way. The samples are summarised in Table 6.

The hydrogels were subjected to microscopic observation, followed by a swelling test and thermal analysis (thermogravimetry and differential scanning calorimetry) to select a suitable matrix and then to assess the effect of the fibre addition on the matrix and its water storage capacity. Wool hydrogels were additionally subjected to a soil water retention test under laboratory conditions.

The dried hydrogels were examined by microscopic observation with a digital microscope (Keyence VHX-900F, Mechelen, Belgium) and a scanning electron microscope (Apreo 2, ThermoFisher Scientific). The swelling test was carried out by immersing dried hydrogels of known weight in water for 48 h (without wool) and 7 days (with wool). The swollen samples were weighed regularly after being gently drained of external water. Measurements were repeated three times for each sample. Based on the collected data, the degree of swelling (SD) was calculated using equation [38,39]:

(1) $$SD=\frac{W_{s}-W_{d}}{W_{d}}$$

where W_s_ is the mass of swollen hydrogel [g] and W_d_ is the mass of dry hydrogel [g]. The equilibrium swelling degree (ESD) was determined when the measured W_s_ reached a constant value.

Thermogravimetric analysis (TG) was performed on swelled hydrogels (TGA 550 Discovery with TRIOS software (Version 5.8.1.14), TA Instruments, New Castle, DE, USA) in platinum crucibles, under a nitrogen and air atmosphere (flow rate: 40 mL/min). The heating rate was 10 °C/min. The study was carried out in the temperature range of 40–600 °C. The mass of the samples was approximately 7 mg. Differential scanning calorimetry (DSC) was performed on swelled hydrogels (DSC 1 with STARe software (Version 16.40), Mettler Toledo, Greifensee, Switzerland). The samples were subjected to a heating (10 °C/min) program in the temperature range of −30–150 °C, under a nitrogen atmosphere, with a flow rate of 30 mL/min. Samples were placed in pierced and sealed aluminium pans. The reference sample was an empty crucible. The mass of the samples was approximately 5 mg. Based on the DSC results and the degree of swelling, the amount of freezing and non-freezing water in the hydrogels was calculated using the following equations [38,39]:

(2) $$W_{\infty }=\frac{ESD}{ESD+1}\cdot 100$$

where W_∞_ is the equilibrium water content of the hydrogel [%];

(3) $$W_{nf}=W_{\infty }-W_{f}$$

where W_nf_ is non-freezing water content [%] and W_f_ is freezing water content [%];

(4) $$W_{f}=\frac{∆H_{endo}}{∆H_{w}}\cdot 100$$

where ∆H_endo_ is the area under the corresponding peak from water melting on the DSC curve and ∆H_w_ is the heat of fusion of pure water (333.3 J/g [40]). TOPEM analysis of the samples with wool was performed on the same apparatus. The samples were subjected to heating (1 °C/min) with modulation amplitude at ±0.5 °C in the temperature range of −30–30 °C.

The water retention test was based on mixing dried hydrogels with dried soil (1 g hydrogel/100 g soil) and adding water (1 mL water/3 g soil). Containers with soil prepared in this way with ventilation available were left at room temperature for 3 weeks and weighed regularly. Measurements were repeated three times for each sample. The water retention W_r_ [%] was calculated using the following equation [18]:

(5) $$W_{r}=\frac{W_{i}-W_{0}}{W_{s}-W_{0}}\cdot 100$$

where W_0_ is the weight of dry soil mixed with hydrogel in a container, W_s_ is the weight of this mix with added water at the start of the experiment, and W_i_ is the weight of this mix on a given day.

## 4. Conclusions

The study confirms the potential for the addition of waste wool fibres as a safe and effective material to enhance water retention in soil. Wool plays a crucial role in shaping the structure of the hydrogel matrix. Its presence helps to maintain open spaces inside the hydrogel matrix, which is shown to be a property essential for creating materials capable of water storage. The performance of hydrogels is closely linked to their net structure because hydrogels with overly dense cross-linked networks are not capable of repeatedly swelling and releasing water. The most important conclusions are as follows:

- Too high a concentration of cross-linker results in a compact hydrogel structure that is unable to swell repeatedly;
- Wool fibres disrupt the structure of the hydrogel network, resulting in weaker swelling, but the beneficial release of water into the soil lasts longer with a high fibre addition;
- Thermal analysis and, in particular, differential scanning calorimetry (DSC) results allow the performance of the material to be accurately predicted and the bonding of the water to the material to be determined. Thermal analysis has shown that, due to the large addition of fibres, the evaporation of water from the hydrogel is impaired, and the process starts at higher temperatures. DSC studies have determined how water is bound in hydrogels. The addition of fibres causes the ratio of freezing and non-freezing water to change. For a large fibre addition, the amount of freezing water is 3 times higher compared to the hydrogel matrix. This can be related to soil water retention results, where the same hydrogels with a high fibre addition (24 and 35%) performed almost 1.5 times better than the hydrogel alone, and 2 times better than the reference sample (soil).

In the long term, incorporating wool not only improves water retention, but can also enrich the soil with nutrients through the gradual biodegradation of the fibres. Properly selected concentrations of hydrogel and wool may be a promising route towards creating a fertiliser material with additional retention-enhancing properties. In the future, it can be an interesting strategy to address agricultural challenges related to climate change and droughts, although further research is needed on the degradation of the material and its long-term effect on the soil.

## Figures and Tables

**Table 1:** Results * of TG analysis for obtained SA hydrogel matrix.

Atmosphere	Sample	T_1%_ [°C]	T_3%_ [°C]	T_5%_ [°C]	T_20%_ [°C]	T_50%_ [°C]	W_r_ [%]	T_1,DTGmax_	T_2,onset_	T_2,DTGmax_	T_3,onset_	T_3,DTGmax_
N_2_	4%SA_01Ca	35	38	42	61	84	1.93	92	210	219	-	-
	6%SA_01Ca	32	32	33	43	58	3.44	66	211	222	-	-
	8%SA_01Ca	35	38	42	60	84	3.09	93	207	215	-	-
	4%SA_05Ca	39	40	40	42	51	16.91	<40	207	220	-	-
	6%SA_05Ca	40	40	40	43	211	26.27	<40	203	208	-	-
	8%SA_05Ca	40	40	40	43	196	25.04	<40	200	202	-	-
Air	4%SA_01Ca	40	40	40	44	53	3.90	50	212	222	541	546
	6%SA_01Ca	40	40	41	51	66	2.43	70	214	220	558	568
	8%SA_01Ca	40	40	42	54	71	1.67	79	210	270	509	514
	4%SA_05Ca	40	40	42	58	204	12.86	50	197	202	523	528
	6%SA_05Ca	40	40	42	64	235	14.03	41	193	195	530	531
	8%SA_05Ca	40	41	43	63	237	15.18	42	194	197	523	529

* T_1(3,5,20,50)%_—temperature at 1, 3, 5, 20, 50% mass loss. W_r_—char residue at 600 °C. T_1(2,3),DTGmax_—1st/2nd/3rd degradation stage. T_2(3),onset_—beginning of 2nd/3rd degradation stage.

**Table 2:** Results of TG analysis for SA hydrogels/wool composites.

Atmosphere	Sample	T_1%_ [°C]	T_3%_ [°C]	T_5%_ [°C]	T_20%_ [°C]	T_50%_ [°C]	W_r_ [%]	T_1,DTGmax_	T_2,onset_	T_2,DTGmax_	T_3,onset_	T_3,DTGmax_
N_2_	6%SA_01Ca_10W	40	40	40	45	55	4.00	56	217	223	-	-
	6%SA_01Ca_15W	40	40	40	45	55	6.54	56	215	223	-	-
	6%SA_01Ca_24W	40	40	40	50	232	21.20	50	208	220	-	-
	6%SA_01Ca_35W	40	40	42	56	248	21.99	<40	210	223	-	-
	6%SA_01Ca_10WK	40	40	40	40	46	11.12	<40	215	222	-	-
	6%SA_01Ca_35WK	40	40	42	61	273	22.55	<40	212	240	-	-
	WOOL	54	79	133	280	342	25.05	48	232	313	-	-
Air	6%SA_01Ca_10W	40	41	42	51	66	1.89	73	214	223	522	526
	6%SA_01Ca_15W	40	41	43	56	74	1.63	83	219	227	528	532
	6%SA_01Ca_24W	40	43	46	64	88	2.84	82	220	273	513	516
	6%SA_01Ca_35W	40	42	44	62	93	5.46	86	211	273	520	525
	6%SA_01Ca_10WK	40	42	44	58	77	1.28	87	213	223	522	526
	6%SA_01Ca_35WK	40	41	43	56	83	1.96	66	213	279	526	529

**Table 3:** Results * of DSC analysis and water states calculation.

Sample	T_m,s_ [°C]	T_m,e_ [°C]	H_endo_ [J/g]	ESD [−]	W_∞_ [%]	W_f_ [%]	W_nf_ [%]
4%SA_01Ca	1	13	247.01	7.9 ± 0.5	88.7 ± 0.6	74.1	14.6 ± 0.6
6%SA_01Ca	−1	12	270.24	46.9 ± 2.1	97.9 ± 0.1	81.1	16.8 ± 0.1
8%SA_01Ca	−1	12	251.54	38.7 ± 1.9	97.5 ± 0.1	75.5	22.0 ± 0.1
4%SA_05Ca	0	12	233.74	1.2 ± 0.1	-	-	-
6%SA_05Ca	−2	9	214.37	1.0 ± 0.1	-	-	-
8%SA_05Ca	−2	10	231.02	1.1 ± 0.1	-	-	-

* T_m,s_ and T_m,e_—temperature of start and end of melting of frozen water. ESD—equilibrium swelling degree. W_∞__/f/nf_—equilibrium/freezing/non-freezing water content of the hydrogel.

**Table 4:** Results of DSC analysis and water states calculation for SA hydrogel/wool composites.

Sample	T_m,s_ [°C]	T_m,e_ [°C]	H_endo_ [J/g]	ESD [−]	W_∞_ [%]	W_f_ [%]	W_nf_ [%]
6%SA_01Ca_10W	−2	13	292.25	16.2 ± 0.7	94.2 ± 0.2	87.7	6.5 ± 0.2
6%SA_01Ca_15W	−4	11	252.75	11.9 ± 0.5	92.3 ± 0.3	75.8	16.4 ± 0.3
6%SA_01Ca_24W	2	15	248.57	7.6 ± 0.3	88.3 ± 0.4	74.6	13.7 ± 0.4
6%SA_01Ca_35W	−1	13	119.06	2.6 ± 0.1	71.9 ± 1.0	35.7	36.2 ± 1.0
6%SA_01Ca_10WK	0	14	266.77	13.7 ± 0.6	93.2 ± 0.3	80.0	13.2 ± 0.3
6%SA_01Ca_35WK	−1	11	96.7	1.4 ± 0.1	58.0 ± 1.1	29.0	29.0 ± 1.1

**Table 5:** Types of hydrogels prepared without wool for matrix selection.

Sample Name	SA Solution [%]	CaCl_2(aq)_ Concentration [M]
4SA_01Ca	4	0.1
6SA_01Ca	6	
8SA_01Ca	8	
4SA_05Ca	4	0.5
6SA_05Ca	6	
8SA_05Ca	8	

**Table 6:** Types of hydrogels prepared with wool fibres.

Sample Name	SA + Wool Solution [% wt.]	CaCl_2(aq)_ Concentration [M]	Wool Fibres [% wt.] *	Type of Milling
6SA_01Ca_10W	6	0.1	10	cutting
6SA_01Ca_15W			15	cutting
6SA_01Ca_24W			24	cutting
6SA_01Ca_35W			35	cutting
6SA_01Ca_10WK			10	cryogenic
6SA_01Ca_35WK			35	cryogenic

* wool fibre concentration in comparison to SA + wool mix without water.

## Data Availability

The original contributions presented in this study are included in the article. Further enquiries can be directed to the corresponding author.
